# Chemical weathering rates and controlling mechanisms of glacial catchments within different climate regimes in the Tibetan Plateau

**DOI:** 10.7717/peerj.15594

**Published:** 2023-07-05

**Authors:** Xiao Guo, Zimiao Zhao, Wenjing Liu, Huiguo Sun, Zhifang Xu

**Affiliations:** 1 Key Laboratory of Cenozoic Geology and Environment, Institute of Geology and Geophysics, Chinese Academy of Sciences, Beijing, China; 2 Center for Excellence in Life and Paleoenvironment, Chinese Academy of Sciences, Beijing, China; 3 University of Chinese Academy of Sciences, Beijing, China

**Keywords:** River chemistry, Yarlung Tsangpo river basin, Glacial catchment, Chemical weathering, Monte Carlo inversion model

## Abstract

**Background:**

Continental weathering plays an important role in regulating atmospheric CO_2_ levels. Chemical weathering in glacial areas has become an intensely focused topic in the background of global change compared with other terrestrial weathering systems. However, research on the weathering of the glacial areas in the Yarlung Tsangpo River Basin (YTRB) is still limited.

**Methods:**

In this article, the major ions of the Chaiqu and Niangqu catchments in the YTRB have been investigated to illustrate the chemical weathering rates and mechanisms of the glacier areas in the YTRB.

**Results:**

Ca^2+^ and HCO${}_{3}^{-}$ dominate the major ions of the Chaiqu and Niangqu rivers, accounting for about 71.3% and 69.2% of the TZ^+^ of the Chaiqu (the total cations, TZ^+^ = Na^+^ + K^+^ + Ca^2 +^ + Mg^2+^, in µeq/L), and about 64.2% and 62.6% of the TZ^+^ of the Niangqu. A Monte Carlo model with six end-members is applied to quantitatively partition the dissolved load sources of the catchments. The results show that the dissolved loads of the Chaiqu and Niangqu rivers are mainly derived from carbonate weathering (accounting for about 62.9% and 79.7% of the TZ^+^, respectively), followed by silicate weathering (about 25.8% and 7.9% of the TZ^+^, respectively). The contributions of precipitation and evaporite to the Chaiqu rivers are about 5.0% and 6.2%, and those to the Niangqu rivers are about 6.3% and 6.2%. The model also calculated the proportion of sulfuric acid weathering in the Chaiqu and Niangqu catchments, which account for about 21.1% and 32.3% of the TZ^+^, respectively. Based on the results calculated by the model, the carbonate and silicate weathering rates in the Chaiqu catchment are about 7.9 and 1.8 ton km^−2^ a^−1^, and in the Niangqu catchment, the rates are about 13.7 and 1.5 ton km^−2^ a^−1^. The associated CO_2_ consumption in the Chaiqu catchment is about 4.3 and 4.4 × 10^4^ mol km^−2^ a^−1^, and about 4.3 and 1.3 × 10^4^ mol km^−2^ a^−1^ in the Niangqu catchment. The chemical weathering rates of the glacier areas in the YTRB show an increasing trend from upstream to downstream. Studying the weathering rates of glacier catchments in the Tibetan Plateau (TP) reveals that the chemical weathering rates of the temperate glacier catchments are higher than those of the cold glacier catchments and that lithology and runoff are important factors in controlling the chemical weathering of glacier catchments in the TP. The chemical weathering mechanisms of glacier areas in the YTRB were explored through statistical methods, and we found that elevation-dependent climate is the primary control. Lithology and glacial landforms rank second and third, respectively. Our results suggest that, above a certain altitude, climate change caused by tectonic uplift may inhibit chemical weathering. There is a more complex interaction between tectonic uplift, climate, and chemical weathering.

## Introduction

As one of the critical Earth system processes, chemical weathering consumes and stores CO_2_ derived from the atmosphere. This process is an important negative mechanism that can balance the CO_2_ degassing from the earth’s interior (mid-ocean ridge and plate subduction) over geological time scales, and it plays an important role in regulating atmospheric CO_2_ levels (Berner, 1991; Walker, Hays & Kasting, 1981). The variation of chemical weathering rates has a profound impact on the climate (Berner & Caldeira, 1997). Li et al. (2022) pointed out that contemporary chemical weathering rates in the glacial areas are ∼3 times higher than that 20 years ago, and are ∼4 times higher than that in major non-glacial areas. Therefore, under current climate change, chemical weathering in the glacial areas is becoming increasingly significant.

The Yarlung Tsangpo River, the highest river in the world (Shi et al., 2011), is located in the Tibetan Plateau (TP), which has the largest store of ice outside the polar regions (Qiu, 2008). From upstream to downstream, the glaciers transition from cold to temperate (Shijin, Yanjun & Yanqiang, 2021; Wang & Ding, 2017). Numerous studies on the weathering have been conducted in the Yarlung Tsangpo River Basin (YTRB) (Hren et al., 2007; Jiang et al., 2015; Liu et al., 2019; Singh, Sarin & France-Lanord, 2005; Yan et al., 2022; Yu et al., 2021c). This results from Raymond’s hypothesis that the uplift of the TP led to the global cooling during the Cenozoic (Raymo & Ruddiman, 1992). However, research on the weathering in the YTRB’s glacial areas is still limited. Previous studies have primarily focused on temperate glacier areas in the southeastern TP and the Himalayas (Biswal et al., 2022; Hasnain, Subramanian & Dhanpal, 1989; Hasnain & Thayyen, 1996; Hodson et al., 2002; Jiang et al., 2018; Li et al., 2019; Liu et al., 2022; Shukla et al., 2018; Singh, Keshari & Ramanathan, 2020; West et al., 2002; Zhang et al., 2021). Few studies have been carried out in the TP’s cold glacier areas (Wu, 2018; Yu et al., 2019; Yu et al., 2021a; Yu et al., 2021b), and there is a lack of research on the chemical weathering mechanisms of glacial areas in the YTRB.

Chemical weathering products are transported into rivers by surface hydrology flow. So as the collector of weathering products, hydrochemistry of rivers is widely used to estimate the weathering rate. Due to the complexity of the weathering process, many factors could influence this process, and there may be interactions between the controlling factors. Some studies have revealed how lithology and land use types affects weathering (Hakim et al., 2021; Hartmann et al., 2014; Zhang et al., 2021), whereas others have emphasized the impact of climate and tectonics (Gernon et al., 2021; West, Galy & Bickle, 2005). Therefore, it has always been challenging to understand the relationships between controlling factors and to clarify the relative importance of each controlling factor in the study of weathering mechanisms. In contrast to large basins, small catchments with a simple geological and climatic background are convenient for critical parameter quantification, which has advantages in the study of the weathering mechanisms (Gaillardet et al., 1999; West et al., 2002). Bufe et al. (2022) found that carbonate weathering is impacted by denudation rate, while silicate weathering is not, based on the investigations of small catchments in the eastern TP. Zhang et al. (2021) proposed that silicate weathering was inhibited by low temperatures while a large amount of freshly ground rock debris produced by glacial erosion enhanced carbonate weathering in the Karuxung River catchment. Jiang et al. (2018) revealed that lithology, climatic factors, and physical erosion rate are the critical parameters controlling chemical weathering rate by studying the small catchments of the Gongga Mount. A following up study by Liu et al. (2022) proposed a strong sulfric acid involvement in chemical weathering and associated CO_2_ budgets on the Tibetan Plateau from the perspective of small catchment. The study on the chemical weathering of glacier areas in the YTRB *via* small catchments contributes to understanding the weathering mechanisms of glacier areas in this region.

Considering that the climate regime of the YTRB changes from a semi-arid climate in the upstream to a humid climate in the downstream (Liu et al., 2013b; Ren, Yao & Xie, 2016) and the distribution of cold and temperate glaciers (Shijin, Yanjun & Yanqiang, 2021), the Chaiqu and Niangqu glacier catchments located at the upper and lower reaches of the river basin were selected to study the chemical weathering mechanisms of the glacial areas in the YTRB. This work aims to (1) quantitatively partition the sources of dissolved load in the glacial catchments; (2) calculate the weathering rates and associated CO_2_ consumption; (3) reveal the key controlling factors and chemical weathering mechanisms of the YTRB’s glacial areas.

## Materials & Methods

### Study area

Chaiqu is located in Zhongba County, southwestern TP. It originates from the Gangdise Mountains and flows into the main stream of the YTRB from the left bank. The topography is high in the north and low in the south, with an average elevation of above 5,000 m. Due to the Himalayas’ blockage of moisture, the climate here is primarily semi-arid. Winter, spring, and summer receive the most precipitation. The annual precipitation and annual mean temperature are 157 mm and 3.2 °C, respectively (Ren, Yao & Xie, 2016). Based on the glacier classification, the glaciers in the Chaiqu catchment are cold glaciers (Shijin, Yanjun & Yanqiang, 2021; Wang & Ding, 2017).

Niangqu is located in Gongbo’gyamda County, Nyingchi, southeastern TP. This river rises from the southwest side of the Nyainqêntanglha Mountains and is a tributary on the left bank of Niyangqu (a tributary of the YTRB). In general, the terrain is high in the north and low in the south, with an average elevation of about 4,930 m. Due to the influence of the South Asian monsoon, this area is characterized by a humid climate and receives the most precipitation from May to September. The annual precipitation is about 674.4 mm and the highest and lowest temperatures occurred in June (15.9 °C) and January (0.6 °C) (Liu et al., 2013b), respectively. The glacial type of Niangqu is the maritime (temperate) glacier (Shijin, Yanjun & Yanqiang, 2021).

**Figure 1:**
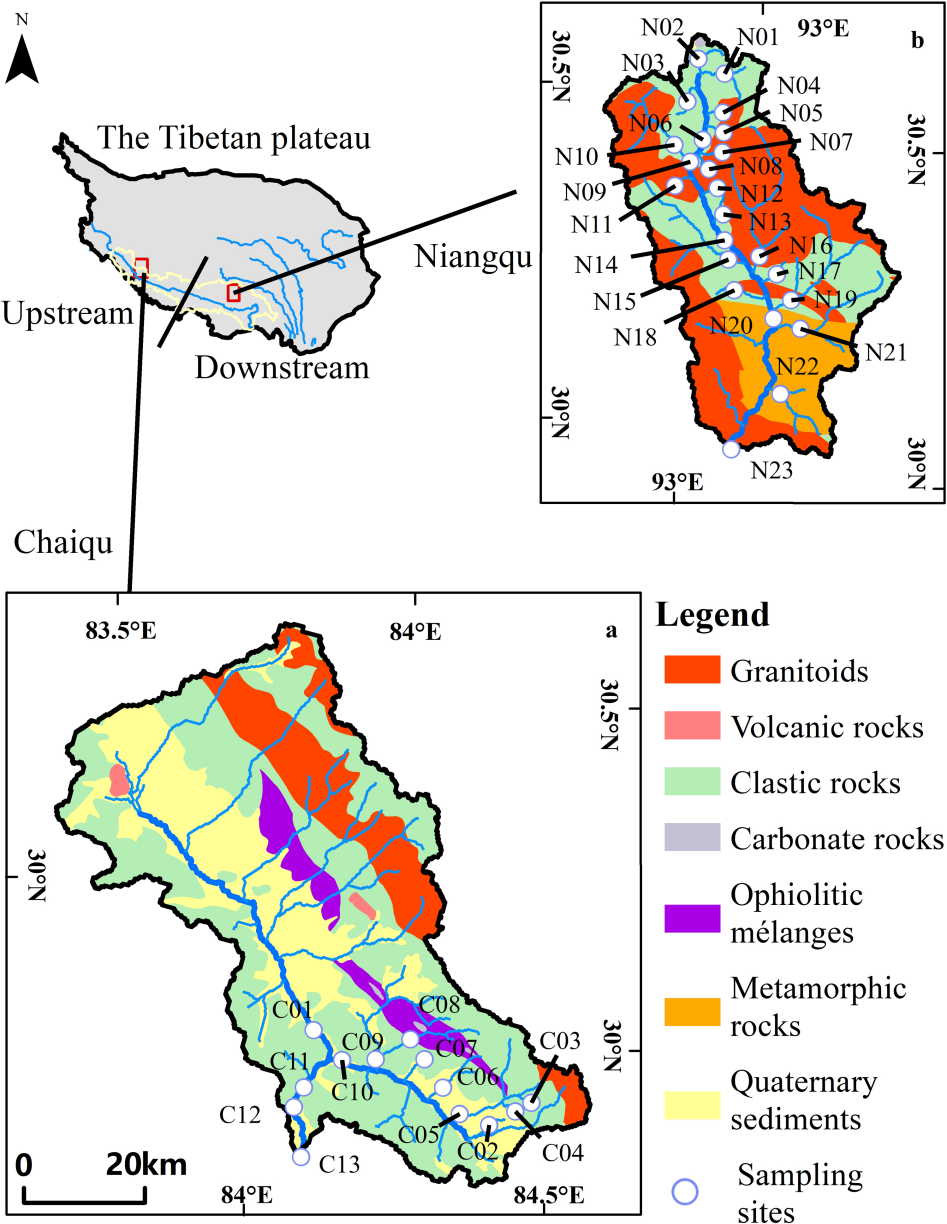
Geological maps and sampling sites. (a) Geological map of Chaiqu, (b) geological map of Niangqu. C stands for Chaiqu, and N stands for Niangqu. The geological maps were modified from Yin et al. (2019).

Overall, the Chaiqu catchment (Fig. 1) is distributed along the northwest. Rock types outcropped in this area include igneous rocks, sedimentary rocks, and ophiolitic mélanges. Granitoids compose the majority of the outcropped igneous rocks, which account for about 14.5% of the Chaiqu catchment area. Granitoids are mainly distributed in the northern part of the Chaiqu catchment, with a tiny portion distributed in the southeast. The granitoids in the Chaiqu catchment consist predominantly of monzonitic granite and granodiorite. The outcrop areas of volcanic rocks are confined to the middle and northwest regions of the Chaiqu catchment. The volcanic rocks in the Chaiqu catchment are mainly composed of andesite and basalt. Clastic rocks dominate the outcropped geological bodies in the Chaiqu catchment, accounting for about 49.2% of the catchment area. Their components are mainly shales, sandstones, and pyroclastic rocks, with a small amount of carbonate minerals. The ophiolitic mélanges in the Chaiqu catchment are complex in lithology and predominantly consist of diabase, basalt, pyroclastic rocks, and siliceous rocks. A small amount of carbonate rock remains on the southeast ophiolitic mélange belt. The outcrop area of ophiolitic mélanges accounts for about 5.7% of the total catchment area.

The Niangqu catchment is distributed along the north-south direction and encompasses igneous rocks, sedimentary rocks, and metamorphic rocks. The lithology of the outcropped granitoids and clastic rocks in the Niangqu catchment is basically similar to that in the Chaiqu catchment. However, no volcanic rocks are found in the Niangqu watershed, and there are metamorphic rocks in the southern part of the catchment. The lithology of metamorphic rocks is mainly schist, gneiss, and amphibole, with a small amount of marble. Its protoliths are mudstones, sandstones, and carbonates. The outcrop areas of granitoids, clastic rocks, and metamorphic rocks account for about 49.7%, 32.4%, and 17.6% of the Niangqu catchment, respectively. Regional geological settings are derived from the Geological Map of the People’s Republic of China (southwest) (1:1,500,000) (Yin et al., 2019).

### Sampling and laboratory analysis

In the Chaiqu and Niangqu catchments, respectively, 13 and 23 water samples were collected in May and June 2018. The sample sites are shown in Fig. 1. Each sample was collected near the center of the river cross-section. The pH value, temperature (T) and the alkalinity (HCO${}_{3}^{-}$) of each sample were measured *in situ*. The pH value and temperature were measured by a portable multi-parameter meter(YSI-6920, USA), with resolutions of 0.01 and 0.1 °C, respectively. The alkalinity was determined by titration with 0.01mol/L HCl. A 25 ml glass acid burette (LG091-2, China) with a resolution of 0.01 ml was used to implement this work, and the pH indicator is bromcresol green-methyl red. Each sample was titrated three times, and the average value of the three results was taken. After collection, samples were filtered on-site with Millipore 0.22 µm nitrocellulose membranes. The filtrate was poured into three 50ml polyethylene bottles that had been previously washed with high-purity HCl, then rinsed with ultrapure water and dried. One bottle of filtrate was acidified to pH <2 with double sub-boiling distilled HNO_3_ for cation analysis, and the other two bottles of filtrate were used for anion and aqueous silica (SiO_2_) concentrations analysis, respectively. The aqueous silica (SiO_2_) concentrations were measured by spectrophotometry using the molybdate blue method. Major cations (Na^+^, K^+^, Ca^2+^, Mg^2+^) were determined by an Inductively Coupled Plasma Atomic Emission Spectrometer (ICP-AES) (IRIS Intrepid II XSP; Thermo Fisher Scientific, Waltham, MA, USA) and the precision is within 3%. Anions (Cl^−^, SO${}_{4}^{2-}$) were determined by ionic chromatography (Dionex 120; Thermo Fisher Scientific, Waltham, MA, USA) with a precision of 5%. The measurement of aqueous silica, major cations and anions was conducted in the weathering and hydrochemistry laboratory of the Institute of Geology and Geophysics, Chinese Academy of Sciences.

### Information extraction

The geomography, hydrology and lithology information were extracted for the studied catchemnts. The results are shown in Table 1. The terrain and area information are obtained from the 90 m resolution DEM (digital elevation model) data which is derived from USGS/NASA SRTM data (Jarvis et al., 2008). Here, the relief amplitude is used to represent the steepness of the catchment. This parameter is calculated by dividing the elevation range of the catchment by its area. The catchment elevation is represented by the mean value of DEM data within the catchemet.

**Table 1:** Geographic and geological parameters of the Chaiqu and Niangqu catchments.

Sample	Discharge	Area	Relief amplitude	Elevation	Runoff	Annual mean temperature (AMT)	Vegetation coverage	Pure silicate rocks	Carbonate-silicate rocks
	m^3^/s	km^2^	m/km^2^	m	mm	°C	%	%	%
Chaique									
C01	6.45	2,545	0.6	5,071	79.9	−2.05	54.9	53.0	47.0
C02	0.01	7.7	97.3	5,084	48.0	−1.19	67.0	78.3	21.7
C03	0.03	7.3	80.0	5,361	112.3	−2.64	23.0	0.0	100.0
C04	0.01	1.6	315.3	5,265	56.1	−1.62	75.4	0.6	99.5
C05	0.43	104	9.7	5,409	130.2	−2.65	87.7	15.9	84.1
C06	0.11	44.3	21.1	5,299	77.7	−2.26	99.7	10.3	89.7
C07	0.01	25.4	28.7	4,969	15.0	−0.27	100.0	18.0	82.0
C08	0.26	92.9	12.4	5,202	87.3	−1.74	94.4	0.0	100.0
C09	0.70	321	4.3	5,081	68.5	−1.17	95.6	19.4	80.6
C10	1.87	932	1.6	5,104	63.4	−1.24	82.0	28.1	71.9
C11	8.39	3,605	0.4	5,073	73.4	−1.74	62.3	45.2	54.8
C12	8.40	3,632	0.4	5,071	73.0	−1.73	62.4	44.9	55.1
C13	8.41	3,669	0.4	5,068	72.3	−1.72	62.7	45.0	55.0
Niangqu									
N01	0.20	67.9	17.0	5,201	92.1	−1.09	30.1	10.7	89.3
N02	0.05	29.6	28.6	5,143	57.9	−1.11	76.7	4.0	96.0
N03	0.11	23.7	43.0	5,181	145.6	−1.19	32.2	11.6	88.4
N04	0.11	13.5	88.5	5,184	266.8	−0.98	34.4	58.4	41.6
N05	0.03	2.7	494.6	5,197	359.9	−1.26	22.3	58.0	42.0
N06	0.82	184	7.7	5,131	141.3	−0.89	46.0	14.2	85.8
N07	0.24	27.1	49.4	5,131	277.1	−0.63	37.2	96.9	3.1
N08	0.02	2.9	338.1	5,083	191.8	−0.70	26.0	71.6	28.4
N09	1.17	223	6.8	5,117	165.6	−0.78	46.3	24.4	75.6
N10	0.78	163	8.4	5,153	151.7	−0.91	56.1	71.7	28.3
N11	0.06	6.7	143.9	5,065	294.9	−0.62	46.5	100.0	0.0
N12	0.04	7.5	152.1	5,079	187.9	−0.34	73.2	58.7	41.3
N13	0.06	6.5	178.4	4,933	315.0	0.60	90.3	36.2	63.8
N14	2.86	476	3.9	5,060	189.3	−0.37	56.5	46.6	53.4
N15	2.74	208	8.7	5,025	415.1	−0.13	58.6	44.5	55.5
N16	2.36	234	8.8	5,057	318.1	−0.46	57.2	95.0	5.0
N17	0.15	12.8	113.8	4,947	375.7	0.75	31.0	19.3	80.7
N18	0.65	50.4	31.5	4,841	406.3	0.61	54.0	50.0	50.0
N19	2.63	210	10.3	4,994	395.6	−0.04	53.2	54.0	46.0
N20	11.84	1,229	1.8	5,011	303.8	−0.12	56.7	55.3	44.7
N21	2.31	177	12.0	4,888	411.5	0.82	58.7	6.9	93.1
N22	1.78	97.0	21.0	4,892	578.8	0.96	46.9	24.6	75.4
N23	19.55	1,813	1.4	4,931	340.0	0.45	60.9	49.8	50.2

Due to the remote location of the Chaiqu and Niangqu catchments and weak research foundation, runoff and discharge were calculated from the mean annual runoff field at 0.0625 degree spatial resolution for the years 2016 to 2018 produced by the China Meteorological Administration Land Data Assimilation System (CLDAS). In order to reflect the precipitation intensity of the catchment, the runoff obtained here is the area weighted average. The discharge is calculated by multiplying the mean annual runoff by the catchment area. The annual mean temperature (AMT) and vegetation coverage derived from Resource and Environment Science and Data Center, Institute of Geographic Sciences and Natural Resources Research, CAS. The raster dataset of AMT for the years 2011 to 2015 with 1 km spatial resolution is used here to extract the catchment’s temperature. The AMT values are calculated by averaging the raster values within the catchment. The vegetation coverage was extracted from the remote sensing monitoring data of China’s land use in 2018 with 1 km spatial resolution. Due to the fact that the primary land use types of the catchments corresponding to the sampling sites are vegetation, glaciers, and bare land, and the bare land is covered by snow and ice in the winter and appears when the temperature rises, compared to the glacier areas, which varies with the ambient temperature, the vegetation coverage is more stable and convenient for statistics. Therefore, vegetation coverage is used to study the effect of land use types on chemical weathering in glacier catchments.

Considering that except for granitoids, vlocanic rocks, and Quaternary sediments, ohter geological bodies contain carbonate minerals in the Chaiqu and Niangqu catchments, the lithology is divided into pure silicate rocks and carbonate-silicate rocks.

### Data treatment

In this study, a Monte Carlo inversion model (Torres et al., 2016) is introduced to quantitatively partition the sources of river ions. This method is analogous to the river ion partition method used in the previous study on global silicate weathering (Gaillardet et al., 1999), which relies on numerous repetitive random samplings to obtain approximate solutions. Before using the model to calculate the end-member contributions, a dimensionless treatment is required. The method is to divide the ionic concentration by its total cation concentration (TZ^+^ = Na^+^ + K^+^ + Ca^2+^ + Mg^2+^) in units of charge equivalents. In order to find out the major factors controlling chemical weathering, the principal component analysis has been used here. This method is a statistical technique for reducing the dimensionality of a dataset while preserving as much data information as possible.

## Results

The pH values, water temperatures, and chemical contents of water samples in the Chaiqu and Niangqu catchments are shown in Table 2. The pH values of the Chaiqu catchment vary little, ranging from 7.15 to 7.96, with an average value of 7.48, which indicates that water samples are all mildly alkaline. The temperatures of C03 and C04 in the Chaiqu catchment are relatively low, close to 0 °C, which is affected by glaciers. From upstream to downstream, the sample water temperatures in the Chaiqu catchment show an overall increasing trend, which is also consistent with the spatial distribution of AMT (Table 2). This indicates that water temperatures in the Chaiqu catchment are affected by AMT. The most important cation of the Chaiqu catchment is Ca^2+^, accounting for about 71.3% of the TZ^+^(TZ^+^ = Na^+^ + K^+^ + Ca^2+^ + Mg^2+^, in µeq/L), with an average value of 1,231.2 µmol/L. The second is Mg^2+^, ranging from 142.9 µmol/L to 748.5 µmol/L. (Na^+^ + K^+^) is the smallest proportion in the TZ^+^, which comprises about 9.8% of the TZ^+^. Based on mean values of anion concentrarions, the anions are in the following order: HCO${}_{3}^{-}$ > SO${}_{4}^{2-}\gt $ Cl^−^. Compared with the small glacier catchments at the same latitude on the TP, the content of Chaiqu ions is higher (Yu et al., 2021b).

**Table 2:** The dissolved chemical composition of the Chaiqu and Niangqu catchments.

Sample	Longitude	Latitude	pH	T	Na^+^	K^+^	Ca^2+^	Mg^2+^	Cl^−^	SO${}_{4}^{2-}$	HCO${}_{3}^{-}$	SiO_2_
	Degree			°C	µmol/L							
Chaiqu												
C01	84.07	29.86	7.55	12.1	613.8	42.6	1,169.9	202.7	185.3	395.6	2,554.9	105.5
C02	84.36	29.83	7.52	10.0	150.5	16.6	1,800.7	581.6	76.7	1,357.6	2,513.6	94.4
C03	84.40	29.89	7.15	1.7	189.0	25.3	1,454.1	748.5	72.9	991.0	2,261.0	81.3
C04	84.38	29.87	7.18	0.4	75.4	18.6	1,410.2	538.0	66.8	1,020.2	1,606.5	66.8
C05	84.35	29.86	7.58	7.5	88.4	30.8	1,241.5	174.1	89.5	319.1	2,451.4	267.8
C06	84.27	29.87	7.46	9.5	47.5	25.3	1,271.7	416.8	75.1	228.9	2,558.5	75.4
C07	84.21	29.86	7.45	17.2	56.1	14.5	983.0	568.2	77.9	275.6	2,284.8	126.9
C08	84.15	29.89	7.35	10.6	53.7	18.5	1,221.0	142.9	81.9	420.3	2,114.0	113.0
C09	84.16	29.87	7.58	10.8	754.0	36.4	1,068.3	293.3	161.8	355.4	2,656.7	111.0
C10	84.14	29.87	7.50	11.5	426.8	27.9	1,238.6	181.9	119.6	398.5	2,351.4	109.6
C11	84.04	29.82	7.50	11.6	493.0	52.7	1,219.6	183.8	215.3	176.6	2,571.9	129.6
C12	84.03	29.79	7.47	11.8	446.6	51.8	1,127.1	250.8	213.4	159.6	2,890.1	131.0
C13	84.07	29.71	7.96	12.7	411.9	35.2	799.9	181.1	167.9	224.7	1,620.0	135.8
Niangqu												
N01	92.92	30.56	7.77	12.3	38.7	18.1	550.4	386.5	36.6	337.4	1,380.4	61.8
N02	92.91	30.56	7.73	11.6	39.4	11.1	578.2	537.0	39.2	343.7	1,713.6	66.0
N03	92.91	30.53	7.98	11.7	39.8	8.3	462.6	187.3	36.6	227.5	714.0	43.7
N04	92.94	30.50	8.02	8.8	46.6	23.0	521.7	176.7	36.2	202.0	1,118.6	61.1
N05	92.94	30.48	8.11	8.0	38.7	18.1	487.7	244.3	37.2	177.5	1,213.8	50.7
N06	92.94	30.45	8.42	11.3	42.3	16.2	623.8	463.4	36.5	524.5	1,344.7	61.1
N07	92.94	30.44	8.08	7.1	37.2	13.0	408.6	157.2	35.8	266.3	690.2	47.9
N08	92.93	30.43	8.23	7.2	52.9	13.7	882.2	391.6	35.5	746.2	1,213.8	59.8
N09	92.92	30.42	8.46	11.0	45.8	16.1	565.6	333.8	37.2	325.0	1,213.8	56.3
N10	92.92	30.42	8.38	11.0	61.3	13.3	506.1	214.0	39.7	324.7	856.8	58.4
N11	92.93	30.40	8.32	6.5	34.2	9.3	346.6	108.5	35.3	167.9	571.2	50.0
N12	92.96	30.38	8.29	8.0	35.6	9.3	324.8	137.3	35.7	183.5	595.0	41.0
N13	92.98	30.34	6.90	8.1	39.7	11.1	449.2	130.8	35.8	185.2	809.2	72.3
N14	93.05	30.29	8.29	11.5	77.1	16.5	560.2	276.5	44.2	336.8	1,047.2	59.1
N15	93.05	30.28	8.24	10.0	43.3	14.4	371.8	145.5	38.9	153.9	797.3	50.7
N16	93.07	30.28	8.21	10.9	53.4	12.9	286.9	144.8	38.8	137.2	642.6	59.1
N17	93.09	30.26	8.23	7.5	201.0	15.2	208.9	148.5	104.2	121.3	618.8	56.3
N18	93.09	30.24	8.27	8.8	39.3	14.9	435.7	259.3	36.3	294.8	737.8	53.5
N19	93.12	30.23	8.05	10.6	41.9	11.2	327.5	87.4	37.5	139.3	595.0	57.0
N20	93.12	30.20	7.34	11.2	63.8	24.6	470.9	212.2	43.9	365.8	785.4	56.3
N21	93.13	30.20	7.48	10.3	66.7	17.0	492.2	160.9	46.7	242.3	809.2	54.9
N22	93.13	30.10	8.04	7.0	39.3	23.9	289.6	179.9	36.9	281.6	452.2	47.9
N23	93.08	30.00	7.49	11.6	82.6	19.0	403.2	176.6	45.3	207.0	833.0	56.3

The average pH value of the Niangqu catchment is 8.01. Except for N13, the other samples are all mildly alkaline. Compared with the Chaiqu, the pH values of the Niangqu are generally higher than those of the Chaiqu. The average water temperature in the Niangqu catchment is 9.65 °C, with a range of 6.5 to 12.3 °C. The predominant cation of the Niangqu catchemnt is Ca^2+^, which ranges from 208.9 µmol/L (N17) to 882.2 µmol/L (N08), and makes up roughly 64.2% of the TZ^+^. Mg^2+^ ranks second and comprises nearly 30.4% of the TZ^+^, with an average value of 228.7 µmol/L. According to the chemical composition of samples collected along the Niangqu main channel (N06, N09, N14, N20, and N23), Ca^2+^ and Mg^2+^ show a decreasing trend from upstream to downstream. The proportion of (Na^+^ + K^+^) in the TZ^+^ is the lowest, while the Na^+^ and K^+^ concentrations along the main channel show an opposite trend to that of Ca^2+^ and Mg^2+^. The most important anion is HCO${}_{3}^{-}$, followed by SO${}_{4}^{2-}$. The spatial variations in HCO${}_{3}^{-}$ and SO${}_{4}^{2-}$ concentrations along the main channel are similar to those in Ca^2+^ and Mg^2+^, but not in Cl^−^. The sample’s (Na^+^ + K^+^)/Cl^−^ molar ratios of the Niangqu catchment ranged from 1.2 to 2.2, significantly deviating from 1, which suggests that the dissolve load is less affected by halite.

## Discussion

### Identifying the sources of major ions

**Figure 2:**
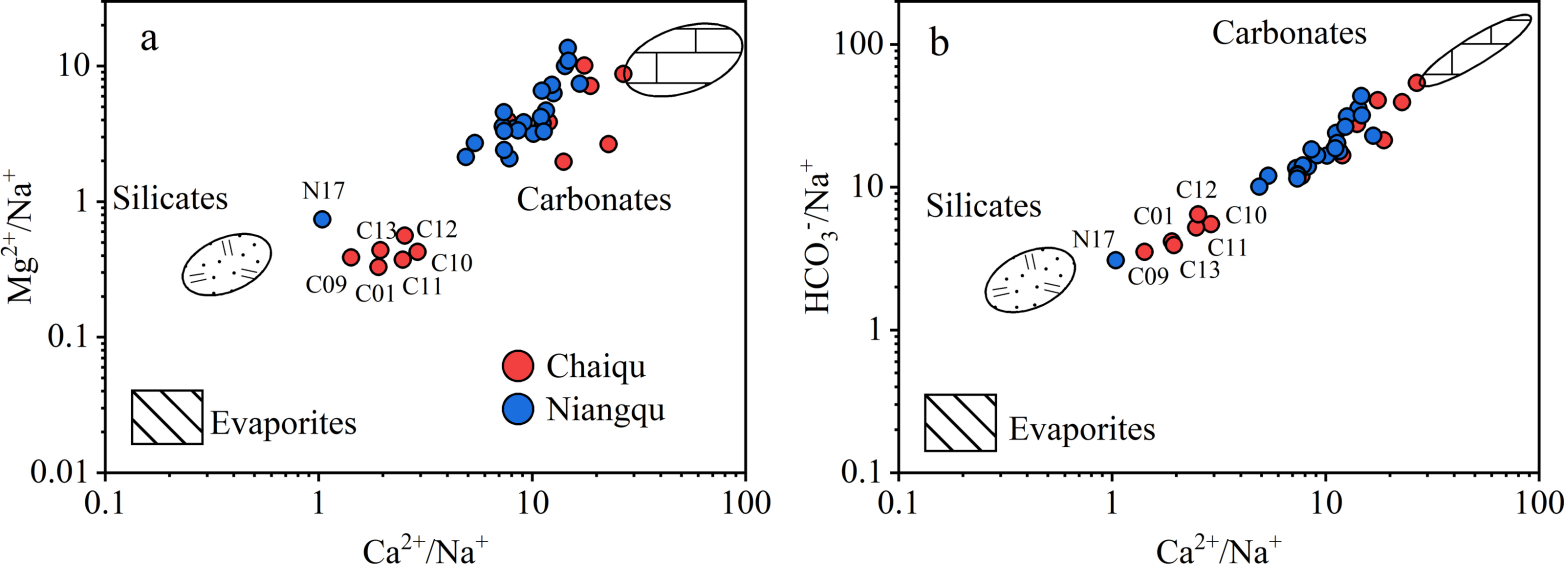
Mixing diagrams using Na-normalized molar ratios. (a) The relationship between Ca^2+^/Na^+^ and Mg^2+^/Na^+^ molar ratios of samples. (b) The relationship between Ca^2+^/Na^+^ and HCO${}_{3}^{-}$/Na^+^ molar ratios of samples.

The dissolved loads of rivers mainly originate from anthropogenic inputs, precipitation, and rock weathering (Gaillardet et al., 1999). Since the Chaiqu and Niangqu catchments are basically in pristine condition, the effect of human activities on river ions is not taken into account. It can be seen from Figs. 2A and 2B that samples in the Chaiqu catchment are concentrated in two areas. The samples along the Chaiqu main channels (C01, C10, C11, C12, and C13) and C09 are close to the silicates, indicating that the silicate weathering has a greater impact on the Chaiqu catchemnt. The rest of the samples in the Chaiqu catchment near the carbonate end-member were collected on tributaries, which are mainly affected by carbonate weathering. Except for N17, the rest of the samples in the Niangqu catchment are greatly affected by the carbonate weathering. The sample distribution deviates downward from the silicate-carbonate line, indicating that both catchments are affected by evaporite dissolution. But the influence is not significant. Some samples of the Chaiqu and Niangqu catchments are not within the range delineated by these three end-members (Fig. 2A), which means that the river ions may be affected by other sources. So, the main sources of river ions in the study areas are precipitation, silicate weathering, carbonate weathering, and evaporite dissolution.

### Precipitation input

Precipitation is an important source of dissolved loads, but the ions it carries are not the products of rock weathering. Therefore, before calculating the weathering rates, the contribution of precipitation to dissolved loads needs to be deducted. Due to the lack of precipitation data in the study areas, the precipitation data in the Tingri and Nyalam counties (Yang et al., 2011) and the Langkazi (Zhang et al., 2021) were used as the precipitation end-member of the Chaiqu catchment. The chemical composition of precipitation (Liu et al., 2013a; Wang et al., 2019) in Sejila Mountain were used to replace the precipitation end-member of the Niangqu catchment. The precipitation end-member ranges of the Chaiqu and Niangqu catchments are shown in Table 3.

**Table 3:** End-members of the Chaiqu and Niangqu.

Limestone	Range of the Chaiqu	Range of the Niangqu	Notes	Dolomite	Range of the Chaiqu	Range of the Niangqu	Notes
Na^+^/TZ^+^	0	0		Na^+^/TZ^+^	0	0	
K^+^/TZ^+^	0	0		K^+^/TZ^+^	0	0	
Ca^2+^/TZ^+^	1	1		Ca^2+^/TZ^+^	0.5	0.5	
Mg^2+^/TZ^+^	0	0		Mg^2+^/TZ^+^	0.5	0.5	
Cl^−^/TZ^+^	0	0		Cl^−^/TZ^+^	0	0	
SO${}_{4}^{2-}$/TZ^+^	0 to 0.5	0 to 0.5		SO${}_{4}^{2-}$/TZ^+^	0 to 0.5	0 to 0.5	
Igneous rocks^a^			Notes	Sedimentary rocks^a^			Notes
Na^+^/TZ^+^	0.07 to 0.40	0.29 to 0.43		Na^+^/TZ^+^	0.01 to 0.15	0.015 to 0.26	
K^+^/TZ^+^	0.02 to 0.73	0.21 to 0.49		K^+^/TZ^+^	0.05 to 0.20	0.003 to 0.45	
Ca^2+^/TZ^+^	0.09 to 0.44	0.05 to 0.27		Ca^2+^/TZ^+^	0.49 to 0.83	0.04 to 0.61	
Mg^2+^/TZ^+^	0.05 to 0.51	0.05 to 0.17		Mg^2+^/TZ^+^	0.11 to 0.24	0.20 to 0.61	
Cl^−^/TZ^+^	0	0		Cl^−^/TZ^+^	0	0	
SO${}_{4}^{2-}$/TZ^+^	0 to 1	0 to 1		SO${}_{4}^{2-}$/TZ^+^	0 to 1	0 to 1	
Precipitation^b^			Notes	Evaporites^c^			Notes
Na^+^/TZ^+^	0.11 to 0.33	0.15 to 0.27		Na^+^/TZ^+^	0.23 to 0.53	0.23 to 0.53	
K^+^/TZ^+^	0.03 to 0.17	0.04 to 0.08		K^+^/TZ^+^	0	0	
Ca^2+^/TZ^+^	0.32 to 0.76	0.55 to 0.77		Ca^2+^/TZ^+^	0.47 to 0.77	0.47 to 0.77	
Mg^2+^/TZ^+^	0.01 to 0.07	0.03to 0.13		Mg^2+^/TZ^+^	0	0	
Cl^−^/TZ^+^	0.11 to 0.39	0.15 to 0.20		Cl^−^/TZ^+^	0.23 to 0.53	0.23 to 0.53	Co-varies with Na^+^/TZ^+^
SO${}_{4}^{2-}$/TZ^+^	0.09 to 0.43	0.05 to 0.14		SO${}_{4}^{2-}$/TZ^+^	0.47 to 0.77	0.47 to 0.77	Co-varies with Ca^2+^/TZ^+^

**Notes.**

a Data cited from Chen et al. (2001), Xiang et al. (2005), Yang et al. (2005), Zhang et al. (2002), Zhang et al. (2003), Zhang et al. (2006), and Zhou et al. (2003).

b Data cited from Liu et al. (2013a), Wang et al. (2019), Yang et al. (2011), and Zhang et al. (2021).

c Data cited from Lerman, Wu & Mackenzie (2007).

### Rock weathering input

The lithology is the main control of river chemistry (Gaillardet et al., 1999). According to geological settings, no evaporites were found in the study areas, which is consistent with the analysis results of Fig. 2. Due to the lack of evaporite data in the study areas, the evaporite mineral content of continental waters is used as a substitute (Lerman, Wu & Mackenzie, 2007). Although the upper crust (2/3 granite and 1/3 basalt) contains NaCl, its content is extremely low. So, this article only focuses on the most prevalent evaporite minerals (halite (NaCl) and gypsum (CaSO_4_^.^H_2_O) or anhydrite (CaSO_4_) in sedimentary rocks (Lerman, Wu & Mackenzie, 2007). Given that the sedimentary rocks in the study areas are primarily composed of sandstones and shales, the halite and gypsum/anhydrite contents of continental waters in sandstones, shales, and evaporites were used as the evaporite end-member values (Table 3).

Based on the geological settings of the Chaiqu and Niangqu catchments, clastic rocks, ophiolitic mélanges, and metamorphic rocks all contain carbonate minerals, and the analysis of river chemistry also shows the effect of carbonate weathering on river ions. Since the main chemical components of carbonate minerals are CaCO_3_ and CaMg(CO_3_)_2_, only these two types of carbonate weathering are considered in this article, and two end-members of carbonate rocks are set in both the Chaiqu and Niangqu catchments. According to these two chemical components, two end-members are termed limestone and dolomite, respectively, and the end-members’ ranges are specified by Eqs. (1) and (2) (Table 3).

(1) \begin{eqnarray*}& {\mathrm{CaCO}}_{3}+{\mathrm{H}}_{2}\mathrm{O}+{\mathrm{CO}}_{2}={\mathrm{Ca}}^{2+}+2{\mathrm{HCO}}_{3}^{-}\end{eqnarray*}

(2) \begin{eqnarray*}& \mathrm{CaMg}({\mathrm{CO}}_{3})_{2}+2{\mathrm{H}}_{2}\mathrm{O}+2{\mathrm{CO}}_{2}={\mathrm{Ca}}^{2+}+{\mathrm{Mg}}^{2+}+4{\mathrm{HCO}}_{3}^{-}.\end{eqnarray*}

**Figure 3:**
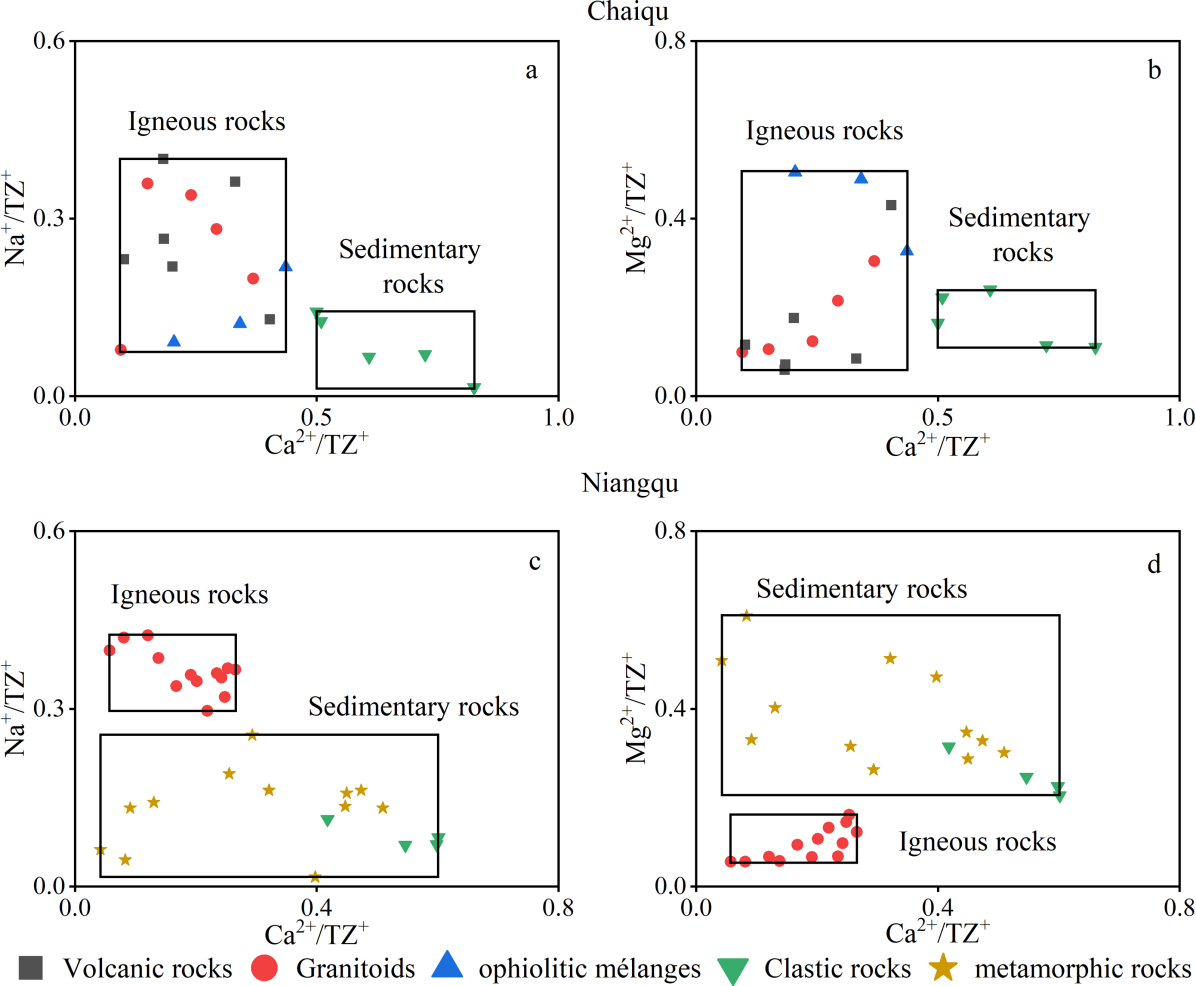
Silicate rock geochemistry scatter plots. (A–B) are the silicate rock geochemistry scatter plot of the Chaiqu catchment. (C–D) are the silicate rock geochemistry scatter plot of the Niangqu catchment. The black rectangles represent the ranges of igneous and sedimentary rock end-members. Note: The clastic rocks and metamorphic rocks in the Niangqu catchment are collectively referred to as sedimentary rocks.

Rocks involved in silicate weathering in the Chaiqu catchment include volcanic rocks, ophiolitic mélanges, granitoids, and clastic rocks. Major element contents of the above rocks were collected, and geochemistry scatter plots (Figs. 3A and 3B) were made. The data of volcanic rocks, ophiolitic mélanges, and granitoids are derived from whole rock major element analysis (Zhang et al., 2003; Zhang et al., 2006; Zhou et al., 2003), and the major element contents of clastic rocks are replaced by the results of stream sediment analysis (Chen et al., 2001). It can be seen from Figs. 3A and 3B that the data points cluster into two groups. The data points of volcanic rocks, ophiolitic mélanges, and granitoids converge, while the data points of clastic rocks are gathered separately. Therefore, two silicate end-members of the Chaiqu catchment are used. The area where the data points of volcanic rocks, ophiolitic mélanges, and granitoids converge is referred to as the igneous rock end-member, and the area where the data points of clastic rocks converge is called the sedimentary rock end-member. The ranges of two end-members are shown in Table 3.

The data points in Figs. 3C and 3D are derived from the major element contents of silicate rocks in the Niangqu catchment. The data of granitoids and metamorphic rocks are obtained from whole rock major element analysis (Xiang et al., 2005; Yang et al., 2005), and the major element contents of clastic rocks are replaced by the results of stream sediment analysis (Zhang et al., 2002). It can be seen from these two figures that the data points are also divided into two areas, so two silicate end-members of the Niangqu catchment are used. The area where the data points of granitoids converge is referred to as the igneous rock end-member. Considering that the protoliths of metamorphic rocks in the Niangqu catchment are sedimentary rocks and the major ion ranges of metamorphic rocks are partially overlaps with those of the clastic rocks in the Niangqu catchment, the area where the data points of metamorphic rocks and clastic rocks converge is collectively referred to as the sedimentary rock end-member. The end-member ranges are shown in Table 3.

Acids involved in rock weathering are mainly carbonic acid, derived from the dissolution of atmospheric CO_2_, or sulfuric acid, formed from the oxidation of sulfide minerals (like FeS_2_). However, rock weathering by sulfuric acid does not consume atmospheric CO_2_; it even releases CO_2_ to the atmosphere. Due to the mild alkalinity of river samples, the case that carbonate weathering by sulfuric acid results in the emission of CO_2_ is not included. Since the possible propotion of carbonate weathering by sulfuric acid ranges from 0 to 1, and the stoichiometric ratio of SO${}_{4}^{2-}$ to the total cations of carbonate in units of charge equivalents is 0.5, the ranges of SO${}_{4}^{2-}$ for carbonate end-members are set 0 to 0.5. For silicate weathering by sulfuric acid, there is only one case. Based on the stoichiometric ratio of SO${}_{4}^{2-}$ to the total cations of silicate in units of charge equivalents, the ranges of SO${}_{4}^{2-}$ for slicate end-members are set 0 to 1.

### Quantitative partitioning of dissolved load sources

After precipitation flows into rivers, the products from end-members will also be carried to the rivers by this process. The chemical composition of river water can be regarded as a mixture of end-members. From a mathematical point of view, the chemical composition of river water is actually a linear combination of the end-members. Therefore, a mixing model is needed to quantitatively partition the dissolved loads. Here, a Monte Carlo model (Torres et al., 2016) is used to simulate this process. The contributions from end-members are yielded by solving the multivariate linear equations. This model is based on the assumption that the contributions of each end-member are greater than or equal to 0, and the sum of all end-member contributions is equal to 1. Through randomly sampling uniform distributions with the ranges of six end-members (Table 3), a total of 500,000 simulations were carried out. The results are shown in Supplemental File, which contains the maximum, minimum, and average values of end-member contributions and proportions of rock weathering by sulfuric acid. In this article, average values are used to represent the end members’ contributions and proportions of rock weathering by sulfuric acid.

### The contributions of end-members to the major ions

**Figure 4:**
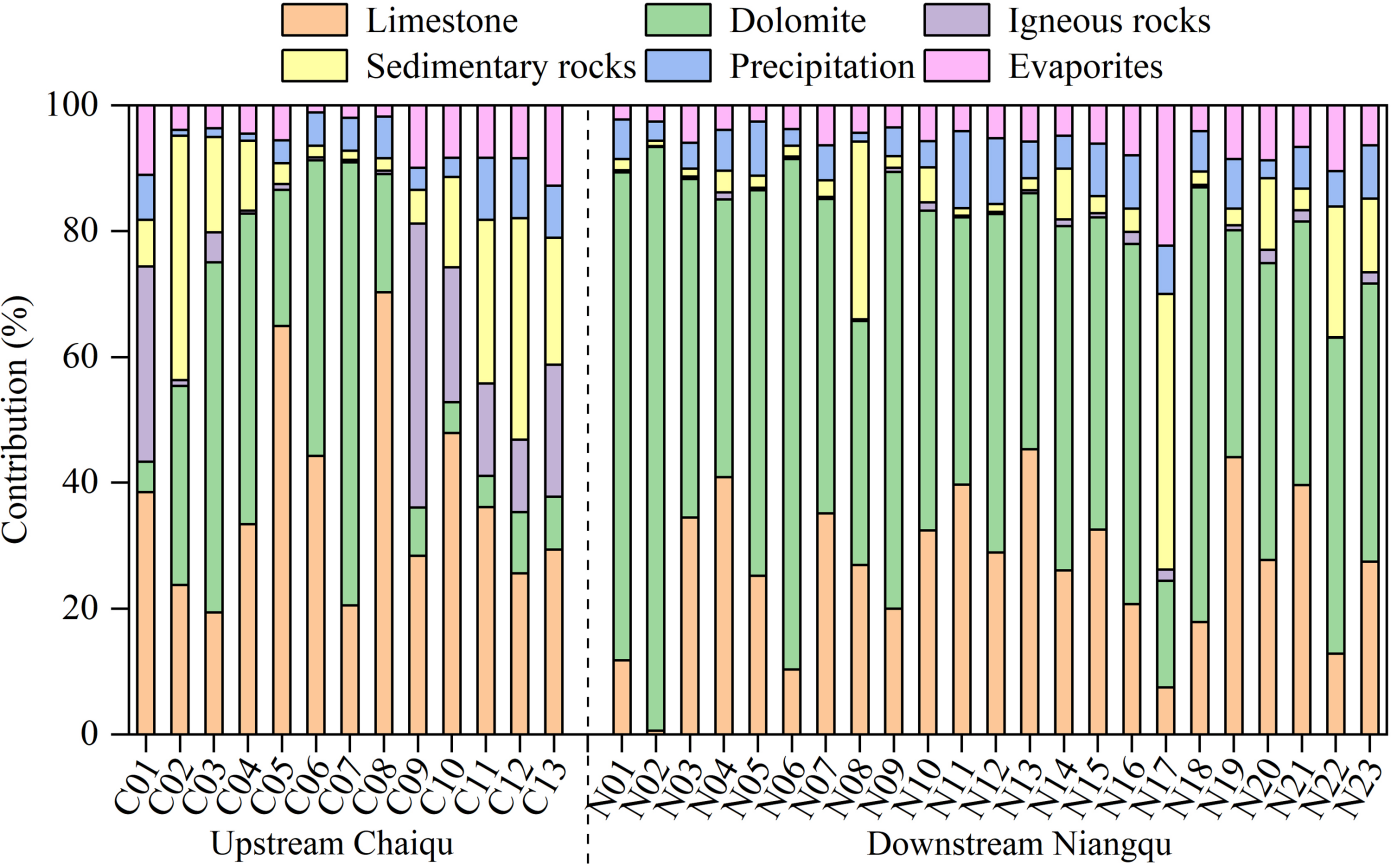
End-members’ contributions (in %) to the TZ^+^ (Na^+^+ K^+^+ Ca^2+^+ Mg^2+^) of river samples.

It can be seen from Fig. 4 that river samples in the Chaiqu catchment are mainly affected by carbonate (limestone + dolomite) weathering, which accounts for 62.8% of the TZ^+^, and the carbonate weathering is dominated by limestone weathering. The contributions of carbonate weathering at C04-C08 are relatively high, which may be related to the outcropped carbonate rocks in the upper reaches of the Chaiqu catchment. The contribution of silicate (igneous rocks + sedimentary rocks) weathering accounts for 25.8% of the TZ^+^, and its contribution is mainly derived from the weathering of sedimentary rocks in the Chaiqu catchment, which accounts for about 14.0% of the TZ^+^. The ophiolitic mélange belt represents the position of the plate boundary, and the clastic rocks distributed on both sides of the belt belong to different plates. According to C02 and C03 sedimentary contributions, it is evident that the contribution of sedimentary rocks from the southern part of the ophiolitic mélange belt is relatively high. Evaporites and precipitation rank third and fourth, respectively, contributing about 6.2% and 5.0% of the TZ^+^. Along the Chaiqu main channels (C01, C10, C11, C12, and C13), the contribution of limestone weathering shows a decreasing trend from upstream to downstream while the contribution of dolomite increases. Overall, the contributions of carbonate weathering in the upstream are higher than those in the downstream, which may be due to the outcropped carbonate rocks in the upper reaches of the Chaiqu catchment. The weathering of sedimentary rocks enhances, whereas the weathering of igneous rocks exhibits a reverse pattern, which is consistent with the distribution of geological bodies in the Chaiqu catchment. The spatial fluctuations of the evaporites and precipitation contributions are stable. Possibly owing to the increase in downstream precipitation (Table 1), the precipitation contributions along the Chaiqu main channel increase slightly from upstream to downstream.

The river ions of the Niangqu catchment are also dominated by carbonate weathering but are primarily derived from dolomite weathering (about 53.2% of TZ^+^). The carbonate contribution at N02 is the largest in the Niangqu catchment, which may be attributed to the outcropped carbonate rocks in this catchment. The silicate weathering contributes approximately 7.9% of the TZ^+^ in the Niangqu catchment, which is dominated by sedimentary rock weathering, and the igneous rock weathering makes a very small impact. The contributions of precipitation and evaporites are close, and their spatial variations are small. The contribution of limestone weathering increases from upstream to downstream, but the contribution of dolomite weathering declines, according to samples taken along the Niangqu main channel(N06, N09, N14, N20, and N23). Overall, the contributions of carbonate show a decreasing trend, which is consistent with the spatial distribution of carbonate-silicate rocks. Due to the higher carbonate mineral content of sedimentary rocks in the upper reaches of Niangqu, weathering contributions from both igneous and sedimentary rocks increase in the lower reaches. Compared with the Chaiqu catchment, the overall contribution of carbonate weathering in the Niangqu catchment is higher. This may be a result of the higher carbonate mineral content in Niangqu geological bodies.

### The contribution of sulfuric acid weathering to the dissolved loads

Based on the model result, the proportions of rock weathering by sulfuric acid were calculated. The algorithm is summing the products of rock end-member SO${}_{4}^{2-}$ contents and their contributions, and the result is expressed as the ratio of SO${}_{4}^{2-}$ to the TZ^+^ in units of charge equivalents. The average, maximum, and minimum proportions are reported in Supplemental File. Here, the samples’ rock weathering proportions driven by sulfuric acid are given as average values.

**Figure 5:**
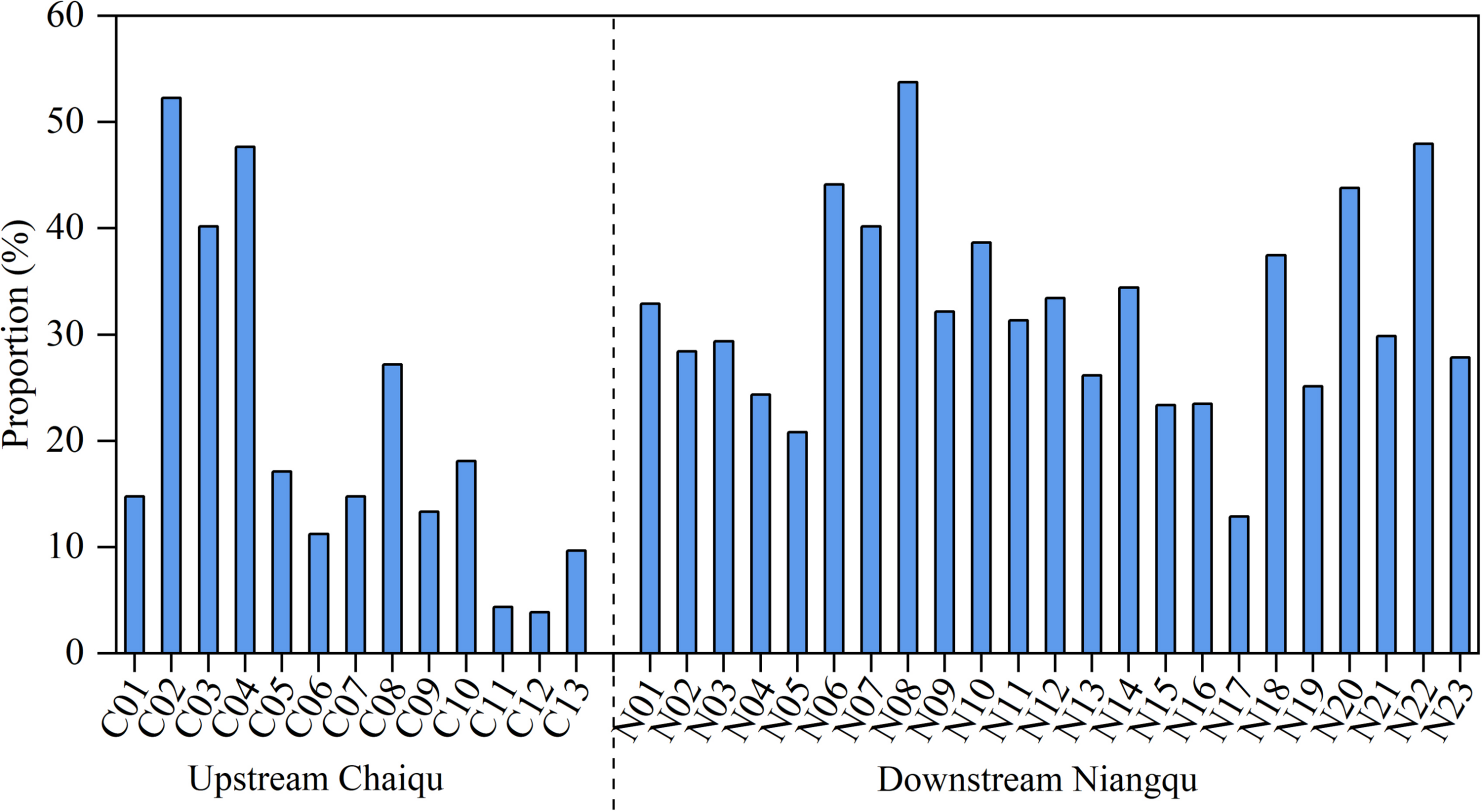
Proportions (in %) of rock weathering by H_2_SO_4_ to the TZ^+^ (Na^+^+ K^+^+ Ca^2+^+ Mg^2+^) of river samples.

It can be seen from Fig. 5 that the sulfuric acid weathering in the Chaiqu catchment ranges from 3.9% (C12) to 52.2% (C02), with an average value of about 21.1%. According to the Chaiqu tributaries (C05, C06, C07, and C09), the proportion of sulfuric acid weathering has varied a little. This may be attributed to the similar geological settings of these catchments. Based on samples collected along the Chaiqu main channels (C01, C10, C11, C12, and C13), the proportions of sulfuric acid weathering exhibit a decreasing trend from upstream to downstream. Considering that sulfide minerals are mainly hosted in organic-rich sedimentary rocks (Torres, West & Li, 2014), the lower proportion of rock weathering by sulfuric acid in the downstream area and the higher proportion of rock weathering by sulfuric acid at C02–C04 may be related to the proportion of carbonate-silicate rocks (Table 1). Compared with the Chaiqu catchment, it can be clearly seen that the proportion of sulfuric acid weathering in the Niangqu catchment is higher, with an average of 32.3%, and fluctuates within a narrower range. This may be due to the different geological periods of sedimentary rocks outcropped in these two catchments. Similar to the Chaiqu catchment, the proportion of sulfuric acid weathering in the Niangqu catchment decreases from upstream to downstream, which is consistent with the distribution of carbonate-silicate rocks in the Niangqu catchment.

The proportions of carbonate and silicate weathering driven by sulfuric and carbonic acids have also been calculated in this article. The results are expressed as the ratios of the charge equivalents of cations produced by carbonate and silicate weathering driven by the weathering agents (sulfuric and carbonic acids) to the total charge equivalents of cations produced by the corresponding rock weathering. Since only sulfuric and carbonic acids are assumed to be weathering agents, the sum of the rock weathering proportions driven by these two acids is equal to 1. Only the proportions of carbonate and silicate weathering driven by sulfuric acid are discussed here.

**Figure 6:**
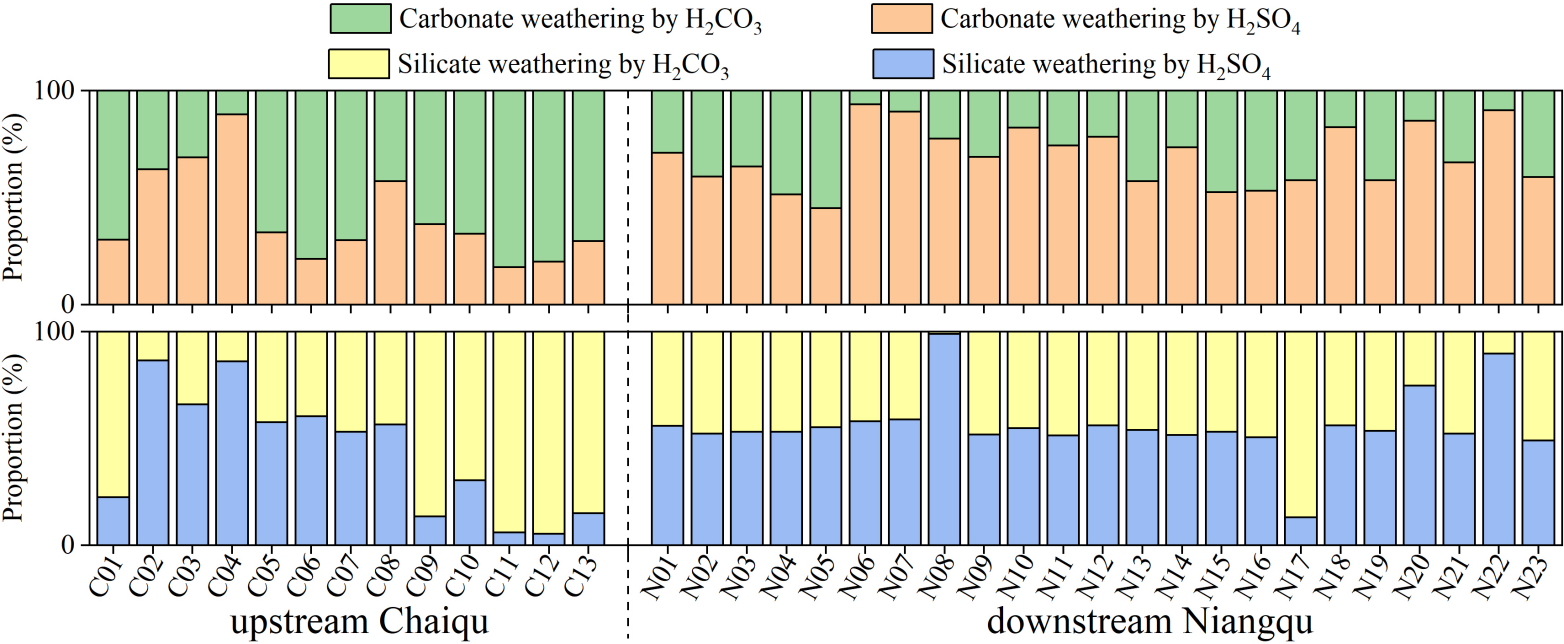
Proportions (in %) of carbonate and silicate weathering driven by H_2_SO_4_ and H_2_CO_3_, respectively.

According to Fig. 6, the proportion of sulfuric acid-driven carbonate weathering ranges from 17.4% (C04) to 88.8% (C11) in the Chaiqu catchment, with an average of about 40.9%. From upstream to downstream, the proportion of sulfuric acid-driven carbonate weathering shows a decreasing trend along the Chaiqu main channels (C01, C10, C11, C12, and C13), and the proportion of sulfuric acid-driven silicate weathering follows a similar spatial trend. This may be related to the decrease in the proportion of carbonate-silicate rocks in the Chaiqu catchment. The proportions of sulfuric acid-driven carbonate and silicate weathering in the Niangqu catchment are about 69.4% and 56.3%, respectively. Along the main channel of the Niangqu catchment, the proportions of sulfuric acid-driven carbonate and silicate weathering shows a decreasing trend from upstream to downstream, but there is little spatial variation in sulfuric acid-driven silicate weathering. Their spatial variation may be affected by the decrease in carbonate-silicate rock proportion. Due to the fact that the protoliths of metamorphic rocks outcropped in the N22 catchment are sedimentary rocks, both the proportions of sulfuric acid-driven carbonate and silicate weathering are relatively high. In the majority of Chaiqu and Niangqu samples, the proportions of sulfuric acid-driven carbonate weathering are greater than the proportions of sulfuric acid-driven silicate weathering. This may be due to the fast dissolution kinetics of carbonate rocks.

### Chemical weathering rates and associated CO_**2**_ consumption

According to the stoichiometry of carbonate and silicate weathering, respectively, driven by carbonic and sulfuric acids, the chemical weathering rates and associated CO_2_ consumption can be calculated (Lerman, Wu & Mackenzie, 2007). The amount of HCO${}_{3}^{-}$ derived from carbonate weathering by sulfuric acid is calculated using the stoichiometry of SO${}_{4}^{2-}$ and HCO${}_{3}^{-}$ (Eq. (1)). Then, based on charge balance, the amount of HCO${}_{3}^{-}$ produced by carbonate weathering by carbonate acide can be found out (Eq. (1)). Finally, according to Eqs. (3) and Eq. (4), the carbonate weathering rate and associated CO_2_ consumption are determined (Yu et al., 2021c). Assuming that all SiO_2_ is derived from silicate weathering, the silicate weathering rate is equal to the sum of cations derived from silicate weathering and SiO_2_ (Eq. (5)) (Zhang et al., 2021). Based on charge balance, the CO_2_ consumption by silicate weathering is calculated.

(3) \begin{eqnarray*}[{\mathrm{HCO}}_{3}^{-}]_{\mathrm{ carb}-\mathrm{sa}}=2[{\mathrm{SO}}_{4}^{2-}]_{\mathrm{ carb}}\end{eqnarray*}

(4) \begin{eqnarray*}[{\mathrm{HCO}}_{3}^{-}]_{\mathrm{ carb}-\mathrm{ca}}=2[{\mathrm{Ca}}^{2+}]_{\mathrm{ carb}}+2[{\mathrm{Mg}}^{2+}]_{\mathrm{ carb}}-2[{\mathrm{SO}}_{4}^{2-}]_{\mathrm{ carb}}-[{\mathrm{HCO}}_{3}^{-}]_{\mathrm{ carb}-\mathrm{sa}}\end{eqnarray*}

(5) \begin{eqnarray*}{\mathrm{TDS}}_{\mathrm{carb}}=([{\mathrm{Ca}}^{2+}]_{\mathrm{ carb}}+[{\mathrm{Mg}}^{2+}]_{\mathrm{ carb}}+1/2[{\mathrm{HCO}}_{3}^{-}]_{\mathrm{ carb}-\mathrm{ca}}+[{\mathrm{HCO}}_{3}^{-}]_{\mathrm{ carb}-\mathrm{sa}})\nonumber\\\displaystyle \quad \times \mathrm{discharge}/\mathrm{area}\end{eqnarray*}

(6) \begin{eqnarray*}{\mathrm{CO}}_{\mathrm{2carb}}=(1/2[{\mathrm{HCO}}_{3}^{-}]_{\mathrm{ carb}-\mathrm{ca}})\times \mathrm{discharge}/\mathrm{area}\end{eqnarray*}

(7) \begin{eqnarray*}{\mathrm{TDS}}_{\mathrm{sil}}=([{\mathrm{Na}}^{+}]_{\mathrm{ sil}}+[{\mathrm{K}}^{+}]_{\mathrm{ sil}}+[{\mathrm{Ca}}^{2+}]_{\mathrm{ sil}}+[{\mathrm{Mg}}^{2+}]_{\mathrm{ sil}}+[{\mathrm{SiO}}_{2}]_{\mathrm{sil}})\times \mathrm{discharge}/\mathrm{area}\end{eqnarray*}

(8) \begin{eqnarray*}{\mathrm{CO}}_{\mathrm{2sil}}=[{\mathrm{HCO}}_{3}^{-}]_{\mathrm{ sil}-\mathrm{ca}}=([{\mathrm{Na}}^{+}]_{\mathrm{ sil}}+[{\mathrm{K}}^{+}]_{\mathrm{ sil}}+2[{\mathrm{Ca}}^{2+}]_{\mathrm{ sil}}+2[{\mathrm{Mg}}^{2+}]_{\mathrm{ sil}}-2[{\mathrm{SO}}_{4}^{2-}]_{\mathrm{ sil}})\nonumber\\\displaystyle \quad \times \mathrm{discharge}/\mathrm{area}\end{eqnarray*}

The subscripts ca and sa represent carbonate acid and sulfuric acid, respectively. [HCO${}_{3}^{-}$]_carb−ca_ and [HCO${}_{3}^{-}$]_carb−sa_ represent bicarbonate derived from carbonate acid weathering and sulfuric acid weathering, respectively.

According to the model’s results and Eqs. (1) to (6), the carbonate and silicate weathering rates and associated CO_2_ consumption were calculated, and their maximum, minimum, and average values are shown in Supplemental File. Table 4 exhibits the average values of weathering rates and CO_2_ consumption. Here, the chemical weathering rates are obtained by dividing the product of the river’s summer ion concentration and annual mean discharge by the catchment area. However, this would underestimate the annual weathering rate. The error is acceptable because: (1) the Yarlung Tsangpo river is a monsoonal river (Ren, Yao & Xie, 2018), and the weathering flux in winter is small; (2) the major ion concentrations vary within a small range in carbonate-dominated catchments, even during flood periods (Gao et al., 2009).

**Table 4:** Chemical weathering rates and associated CO_2_ consumption.

Samples	TDS_carb_	TDS_sil_	Sum	CO_2carb_	CO_2sil_	Sum
	ton km^−2^ a^−1^			10^4^ mol km^−2^ a^−1^		
Chaiqu						
C01	5.9	2.5	8.4	4.2	8.2	12.3
C02	6.3	2.0	8.4	2.1	1.6	3.6
C03	18.5	2.5	21.0	5.7	3.2	9.0
C04	8.9	0.7	9.7	0.9	0.3	1.2
C05	16.5	2.4	18.9	11.0	0.7	11.7
C06	11.9	0.5	12.3	9.6	0.2	9.8
C07	2.1	0.1	2.2	1.5	0.04	1.6
C08	10.8	0.7	11.5	4.6	0.2	4.8
C09	4.3	2.8	7.1	2.8	10.6	13.3
C10	5.5	1.9	7.4	3.8	5.4	9.2
C11	5.1	2.5	7.6	4.5	9.6	14.0
C12	4.2	2.7	6.9	3.7	10.7	14.4
C13	3.3	2.0	5.2	2.5	6.3	8.8
Niangqu						
N01	7.5	0.4	7.9	2.3	0.2	2.5
N02	5.8	0.3	6.0	2.5	0.1	2.5
N03	8.4	0.5	8.8	3.1	0.2	3.2
N04	16.1	1.4	17.5	8.1	0.8	8.9
N05	22.6	1.4	24.0	13.0	0.6	13.6
N06	13.6	0.7	14.2	0.9	0.3	1.2
N07	13.5	1.0	14.5	1.4	0.4	1.7
N08	15.9	3.2	19.1	3.7	0.1	3.8
N09	13.1	0.7	13.8	4.3	0.4	4.6
N10	9.2	0.8	10.1	1.6	0.6	2.3
N11	11.2	1.0	12.2	3.0	0.2	3.2
N12	7.2	0.5	7.8	1.6	0.1	1.7
N13	16.0	1.6	17.5	6.9	0.4	7.3
N14	12.9	1.3	14.2	3.6	1.5	5.1
N15	18.0	1.6	19.6	8.9	0.7	9.6
N16	11.0	1.5	12.5	5.4	0.8	6.3
N17	4.1	4.0	8.1	1.9	13.9	15.7
N18	24.2	1.6	25.8	4.4	0.6	5.0
N19	13.7	1.6	15.3	5.9	0.5	6.4
N20	15.9	2.2	18.1	2.0	1.2	3.2
N21	22.6	2.0	24.6	7.8	1.4	9.2
N22	17.4	3.9	21.2	1.5	1.1	2.6
N23	14.8	2.3	17.0	6.2	3.0	9.2

The carbonate weathering rate of the Chaiqu catchment is about 7.9 ton km^−2^ a^−1^, which is significantly higher than the silicate weathering rate (1.8 ton km^−2^ a^−1^). Due to the high silicate weathering contributions of C01, C09, C10, C11, C12, and C13, the differences in their carbonate and silicate weathering rates are small. Along the Chaiqu’s main channels, both carbonate and silicate weathering rates show a decreasing trend from upstream to downstream. In the Chaiqu catchment, the CO_2_ consumption by carbonate weathering ranges from 0.9 to 11.0 × 10^4^ mol km^−2^ a^−1^. The CO_2_ consumption by silicate weathering is about 4.4 × 10^4^ mol km^−2^ a^−1^, close to that by carbonate weathering. The CO_2_ consumption by silicate weathering at C01, C09, C10, C11, C12, and C13 is higher than that by carbonate weathering, which is related to their silicate weathering contribution. From upstream to downstream, the total CO_2_ consumption by carbonate and silicate weathering shows a decreasing trend, similar with the pattern of rock weathering rates.

The carbonate weathering rate of the Niangqu catchment ranges from 4.1 to 24.2 ton km^−2^ a^−1^, with an average of about 13.7 ton km^−2^ a^−1^. Except for N17, other samples’ carbonate weathering rates are clearly higher than their silicate weathering rates (ranging from 0.3 to 4.0 ton km^−2^ a^−1^). This is attributed to the high silicate weathering contribution of N17. Compared with the Chaiqu catchment, the Niangqu catchment has a higher carbonate weathering rate, while their silicate weathering rates are close. Combined with the chemical weathering rates of the glaciated Karuxung River catchment (Zhang et al., 2021), it can be seen that the chemical weathering rates of the glacier areas in the YTRB show an increasing trend from upstream to downstream. It can be seen from Table 1 that except for runoff and AMT, the other parameters are close, so it is speculated that these two parameters are the main reasons for the difference in weathering rates between the Chaiqu and Niangqu catchments. The carbonate and silicate weathering rates of the Niangqu catchment increase from upstream to downstream, but the contribution of carbonate weathering shows a decreasing trend. This may be connected to the chemical weathering enhancement caused by the increase in runoff and the temperature rise. The CO_2_ consumption by carbonate weathering in the Niangqu catchment is about 4.3 × 10^4^ mol km^−2^ a^−1^, which is close to that in the Chaiqu catchment, while the CO_2_ consumption by silicate weathering is less than that in the Chaiqu catchment. Except for N17, the CO_2_ consumption by carbonate weathering is higher than that by silicate weathering. Although the rock weathering rate of the Niangqu catchment is higher than that of the Chaiqu catchment, its CO_2_ consumption is smaller than that of the Chaiqu catchment, which may be due to the higher proportion of sulfuric acid weathering in the Niangqu catchment.

Since there are many ways to describe the weathering rate of glacier basins in the collected literature, the TCDR (total cationic denudation rate) is used here to represent the weathering intensity. It can be seen from Table 5 that, for the cold glacier catchments, the chemical weathering rate of the Chaiqu catchment is lower than that of the Qiyi glacier in the northeast TP and is also lower than that of the Qugaqie in the central TP. Compared with the temperate glacier catchments in the southeast TP and the south slope of the Himalayas, the chemical weathering rate of the Niangqu catchment is lower. Overall, the chemical weathering rates of the temperate glacier catchments in the southeast TP and the south slope of the Himalayas are higher than those of the cold glacier catchments in the northeast and central TP, showing south-high and north-low features. This may be related to the abundant rainfall and vapor sources in temperate glacier areas (Shijin, Yanjun & Yanqiang, 2021). There is a clear positive correlation between the TCDR and runoff in the TP’s glacier catchments (Table 5), and the weathering rate increases as runoff increases. Comparing the Chaiqu and Qugaqie catchments, which have similar lithologies, shows that the weathering rate of the Qugaqie catchment with high runoff is significantly higher than that of the Chaiqu catchment, indicating that runoff is an important factor controlling the weathering of TP’s glacier catchments. This is because the increase in runoff will provide more fresh minerals, which promote the weathering reaction. By comparing Dokriani Glacier and Qugaqie, which have similar runoff, it can be seen that lithology also has an important impact on the chemical weathering of glacier catchments.

**Table 5:** Chemical weathering rates in the study areas and glacier catchments surrounding the Tibetan plateau.

Glacier types	Location	Basin	Bedrocks	Runoff	TCDR^a^	References
				mm	ton km^−2^ a^−1^	
Cold glaciers	Northeast TP	Qiyi Glacier	Metamorphic rocks	390	10.9	Wu (2018)
		Gangdis Montains	Chaiqu	Granitoids, clastic rocks	72.28	2.6	This study
	Central TP	Qugaqie	Granitoids, clastic rocks	1158	16.4	Yu et al. (2021a), Yu et al. (2021b) and Yu et al. (2021c)
Temperate glaciers	Nyainqêntanglha Mountains	Niangqu	Metamorphic rocks, Granitoids, clastic rocks	339.97	6.5	This study
		Southeast TP	Hailuogou Glacier	Clastic rocks	4503	64.7	Li et al. (2019)
		South slope of the Himalayas	Batal Glacier	Metamorphic rocks, granitoids	–	120.2	Singh, Keshari & Ramanathan (2020)
				Batura Glacier	Carbonate rocks, granitoids	1600	30.9	Hodson et al. (2002)
				Chhota-Shigri Glacier	Metamorphic rocks	3500	17.4	Hasnain, Subramanian & Dhanpal (1989)
	Dokriani Glacier		Metamorphic rocks, granitoids	1120	9.7	Hasnain & Thayyen (1996)

**Notes.**

a TCDR: total cationic denudation rates.

### Control mechanisms of chemical weathering

Chemical weathering is affected by numerous factors, which are roughly divided into climate (temperature and precipitation), topography, land use types, and lithology. In this study, the geographical parameters of the above four types were extracted (Table 1). Comparing the geographical parameters of C13 and N23 reveals that under identical geological and topographic conditions, increases in runoff and temperature accelerate chemical weathering. By comparing the C04 and N02 catchments, which have similar geological and climatic backgrounds, it is clear that the increase in relief amplitude promotes chemical weathering. The N06 and N10 catchments have similar topographic and climatic backgrounds. However, the weathering rates of these two catchments are different, indicating the impact of lithology on chemical weathering.

Because of the complexity and variability of controlling factors and possible interactions between these factors, this will hinder the study of control mechanisms. In order to study the mechanisms of chemical weathering in the glacier catchments. The principal component analysis was used to clarify the relationship between controlling factors and identify principal components. Then, based on the multiple regression equations between principal components and weathering rates, the control mechanisms of chemical weathering were studied.

Since silicate rocks plus carbonate-silicate rocks equal 100%, carbonate-silicate rocks are selected here to study the effect of lithology on chemical weathering. According to the results of principal component analysis (Table 6), the three principal components with the largest eigenvalues were extracted, which explain 82% of the total variance.

**Table 6:** Component matrix of controlling factors.

	Z_1_	Z_2_	Z_3_
Annual mean temperature (AMT)	0.92	0.26	0.18
Runoff	0.88	−0.09	0.20
Elevation	−0.84	−0.41	0.04
Relief amplitude	0.08	−0.75	0.34
Vegetation coverage	−0.38	0.71	−0.08
Carbonate-silicate rocks	−0.46	0.39	0.77

It can be seen that the first principal component (Z_1_) has strong positive correlations with the AMT and runoff and a strong negative correlation with the elevation. In the TP, the AMT of catchments decreases with increasing elevation, so the loadings of these two parameters are opposite. Annual precipitation in the southern TP is dominated by the Indian Summer Monsoon (Hren et al., 2009). Due to the obstruction of the Himalaya, the moisture derived from the Indian Monsoon is transported along the main channel of the YTRB from the eastern syntaxis of the Himalaya or passes through the central Himalaya to the interior of the TP (Hren et al., 2009; Ren, Yao & Xie, 2016; Yu et al., 2016). Therefore, the precipitation in the YTRB is heavily influenced by orography. The characteristic of orographic precipitation is that, above a certain elevation, the precipitation follows a decreasing trend with altitude (Singh & Kumar, 1997). So, in the high-altitude YTRB, the precipitation of the Chaiqu catchment, which has a higher elevation, is less than that of the Niangqu catchment. As a result, the loadings of elevation and precipitation are also opposite. Thus, Z_1_ is an index of the elevation-dependent climate. The second principal (Z_2_) shows a strong positive correlation with the vegetation coverage and a strong negative correlation with the relief amplitude. Beacause the steep terrain is not suitable for soil formation, the area with a large slope has low vegetation coverage. So the loadings of vegetation coverage and relief amplitude are opposite. In the glacier catchments, the land use types in steep terrain areas are mainly glaciers and bare land, and the bare land is covered by snow and ice in winter. So the steep terrain is related to glaciation. Thus, Z_2_ can be regarded as an index of glacial landforms. The third principal component (Z_3_) is positively correlated with carbonate-silicate rocks. Therefore, it reflects lithology information.

(9) \begin{eqnarray*}& {\mathrm{TDS}}_{\mathrm{carb}}=0.426{Z}_{1}-0.275{Z}_{2}+0.310{Z}_{3}+0.000\end{eqnarray*}

(10) \begin{eqnarray*}& {\mathrm{TDS}}_{\mathrm{sil}}=0.308{Z}_{1}-0.035{Z}_{2}+0.048{Z}_{3}+0.000\end{eqnarray*}

Based on the scores of principal components and rock weathering rates, two multiple regression equations between rock weathering (carbonate and silicate weathering) and three components were established (Eqs. (7) and (8)). All the equations passed the Durbin-Watson test and collinearity diagnostics.

According to Eqs. (7) and (8), the elevation-dependent climate (Z_1_) is the first control on the chemical weathering of glacier catchments in the YTRB, indicating that the increases in runoff and temperature caused by the decrease in elevation have the greatest impact on rock weathering rates. The lithology (Z_3_) is the second control on rock weathering rates. According to the results produced by the model (Fig. 4), it can be seen that, except for a few sampling sites, the weathering contributions of sedimentary rocks (clastic and metamorphic rocks) at most sampling sites are higher than those of igneous rocks (granites, volcanic rocks, and ophiolitic mélanges), indicating that sedimentary rocks have weak weathering resistance. Because the pure silicate rocks outcropped in the sampling site catchments are mainly high-weatherability granites, volcanic rocks, and Quaternary sediments, while the outcropped carbonate-silicate rocks are mainly clastic and metamorphic rocks containing carbonate minerals, the increase in the outcropped area of carbonate-silicate rocks will enhance the rock weathering. The index of glacial landforms (Z_2_) ranks third. It is obvious that the steep terrain and glaciers are favorable to fresh mineral exposure and migration, accelerating the rock weathering. Our results suggest, that above a certain altitude, the decrease in precipitation and temperature caused by the increase in altitude limits the chemical weathering of the catchments, revealing a complex relationship between tectonic uplift, climate, and chemical weathering.

## Conclusions

In the Chaiqu and Niangqu catchments, the major cations are dominated by Ca^2+^ and Mg^2+^, accounting for about 90.2% (Ca^2+^ + Mg^2+^) of the TZ^+^ of the Chaiqu and about 94.5% of the TZ^+^ of the Niangqu, followed by Na^+^ and K^+^. HCO${}_{3}^{-}$ is the most important anion, accounting for approximately 69.2% of the TZ^+^ of the Chaiqu and about 62.6% of the TZ^+^ of the Niangqu, followed by SO${}_{4}^{2-}$ and Cl^−^. The river ions are mainly derived from carbonate weathering, while some samples are greatly affected by silicate weathering. A Monte Carlo model with six end-members is used to quantitatively partition the dissolved loads and the proportion of rock weathering by sulfuric acid. The results show that the contributions of carbonate weathering to the Chaiqu and Niangqu catchments are about 62.9% and 79.7%, followed by silicate weathering (about 25.8% and 7.9% of the TZ^+^). The contributions of precipitation and evaporite are low, accounting for about 5.0% and 6.2% of the Chaiqu TZ^+^, and about 6.3% and 6.2% of the Niangqu TZ^+^. The proportion of sulfuric acid weathering in the Chaiqu and Niangqu catchments is about 21.1% and 32.3%, which is higher in the Niangqu catchment.

The carbonate weathering rates of the Chaiqu and Niangqu catchments are about 7.9 and 13.7 ton km^−2^ a^−1^, and their silicate weathering rates are about 1.8 and 1.5 ton km^−2^ a^−1^ respectively. Except for a few samples, CO_2_ consumption by carbonate weathering in the Chaiqu and Niangqu catchments is higher than that by silicate weathering. The chemical weathering rates of the glacier areas in the YTRB increase from upstream to downstream. By comparing the weathering rates of glacier catchments in the Tibetan Plateau (TP), it can be seen that the chemical weathering rates of the temperate glacier catchments are higher than those of the cold glacier catchments and that lithology and runoff are important factors in controlling the chemical weathering of glacier catchments in the TP.

The relationships between controlling factors were clarified by principal component analysis, and three principal components were determined. Based on the multiple regression equations between the three principal components and rock weathering (carbonate and silicate weathering) rates, we found that the primary control on the glacier catchments in the YTRB is elevation-dependent climate. The decrease in temperature and precipitation caused by elevation restrains chemical weathering. The second control is lithology. The increase in outcrop area of carbonate-silicate rocks enhances rock weathering. Glacial landforms rank third. Glaciers and their associated steep terrain promote the exposure of fresh minerals and the migration of weathering products, thereby increasing the rate of rock weathering. Our findings indicate that, above a certain altitude, climate change driven by altitude rise may limit chemical weathering. Thus, the mechanisms between tectonic uplift, climate, and chemical weathering require further elucidation.

##  Supplemental Information

10.7717/peerj.15594/supp-1 File S1 Raw measurements and the Monte Carlo model calculation results All geographical, geological, and chemical parameters.

10.7717/peerj.15594/supp-2 Supplemental Information 2 The statistical reports of principal component analysis and multiple regression analysis

## Additional Information and Declarations

### Data Availability

The following information was supplied regarding data availability: The raw measurements and statistical reports are available in the Supplementary Files.
